# Acceptability and Engagement of a Smartphone-Delivered Interpretation Bias Intervention in a Sample of Black and Latinx Adults: Open Trial

**DOI:** 10.2196/56758

**Published:** 2024-07-31

**Authors:** IreLee Ferguson, Grace George, Kevin O Narine, Amari Turner, Zelda McGhee, Harris Bajwa, Frances G Hart, Sierra Carter, Courtney Beard

**Affiliations:** 1 Division of Depression and Anxiety Disorders McLean Hospital Belmont, MA United States; 2 Department of Basic Neuroscience McLean Hospital Belmont, MA United States; 3 Department of Clinical Psychology William James College Newton, MA United States; 4 Department of Psychology Georgia State University Atlanta, GA United States; 5 Department of Psychiatry McLean Hospital/Harvard Medical School Belmont, MA United States

**Keywords:** interpretation bias, anxiety, depression, Black, Latinx, smartphone, mobile phone

## Abstract

**Background:**

Access to evidence-based interventions is urgently required, especially for individuals of minoritized identities who experience unique barriers to mental health care. Digital mental health interventions have the potential to increase accessibility. Previous pilot studies testing HabitWorks, a smartphone app providing an interpretation bias intervention, have found strong engagement and adherence for HabitWorks; however, previous trials’ samples consisted of predominantly non-Hispanic, White individuals.

**Objective:**

This study conducted an open trial of HabitWorks in a community sample of adults who identified as Black, Hispanic or Latinx, or both. This study aims to test safety, acceptability, and engagement with the HabitWorks app for Black and Latinx adults.

**Methods:**

Black, Hispanic or Latinx adults (mean age 32.83, SD 11.06 y; 22/31, 71% women) who endorsed symptoms of anxiety or depression were asked to complete interpretation modification exercises via HabitWorks 3 times per week for 1 month. Interpretation bias and anxiety and depression symptoms were assessed at baseline and posttreatment assessments. Participants completed qualitative interviews to assess overall perceptions of HabitWorks.

**Results:**

Of the 31 participants that downloaded the app, 27 (87%) used HabitWorks all 4 weeks. On average, participants completed 15.74 (SD 7.43) exercises out of the 12 prescribed, demonstrating high engagement. Acceptability ratings met all a priori benchmarks except for relevancy. Qualitative interviews also demonstrated high acceptability and few negative experiences. Significant improvements were found in interpretation style (t_30_=2.29; *P*<.001), with a large effect size (Cohen *d*=1.53); anxiety symptoms (t_30_=2.29; *P*=.03), with a small effect size (Cohen *d*=0.41); and depression symptoms (t_30_=3.065; *P*=.005), with a medium effect size (Cohen *d=*0.55).

**Conclusions:**

This study adds to the literature evaluating digital mental health interventions in Black and Latinx adults. Preliminary results further support a future controlled trial testing the effectiveness of HabitWorks as an intervention.

## Introduction

### Background

The tendency to resolve ambiguity in a threatening or negative manner, that is, interpretation bias, has been associated with most emotional disorders [1]. HabitWorks is a personalized, transdiagnostic, smartphone-delivered intervention that targets this type of interpretation bias [2]. The primary feature of HabitWorks is a word-sentence association exercise that reinforces users for making benign interpretations and rejecting negative interpretations of ambiguous situations through repeated practice [3]. HabitWorks also includes several features expected to enhance user engagement, such as personalization of the ambiguous situations presented, personally scheduled notifications, performance feedback, level progression, in-app mood monitoring, and a diary feature. In addition, the exercises require only 5 minutes and can be completed at the user’s convenience. HabitWorks showed good feasibility and acceptability in a pilot trial of adults receiving acute psychiatric care [2] and parents youth with anxiety [4], as well as excellent adherence and engagement [5].

This study aims to obtain feasibility and acceptability data about HabitWorks in a sample of adults identifying as Black, Hispanic, or Latinx. The rationale for conducting this pilot study was 2-fold. First, although digital mental health interventions (DMHIs) can overcome the most common barriers to receiving traditional mental health treatment (eg, availability of a provider, cost, transportation, and stigma) [6-9], the DMHI field has not yet realized its potential to increase access to evidence-based interventions. DMHIs are not reaching people who have typically not accessed mental health treatment; most data to date come from White women—the same demographic that is already best served by existing treatments [10,11]. For example, Ellis et al [12] recently reported that 97% of studies in a review of internet-delivered cognitive behavioral therapy either did not include participants of minoritized races and ethnicities or did not report on participant ethnoracial identities. This was further supported in another systematic review of internet-delivered cognitive behavioral therapy trials that found most studies did not report on race and ethnicity or included predominantly non-Hispanic White samples [13]. In a systematic review of culturally adapted DMHIs, only 4 studies examined Black American participants, and none of the studies examined anxiety in the Hispanic or Latinx communities [12]. DMHI developers and researchers must be more intentional to avoid exacerbating the health and access disparities they seek to address.

Second, it is unclear how minoritized groups respond to interpretation bias interventions. Evidence-based treatments may have harmful effects if applied in a one-size-fits-all manner and without attending to potential sociocultural influences. For example, it is generally recommended that therapists not apply cognitive restructuring for interpretations of racist experiences and, instead, focus on the internalized beliefs from these experiences [14]. Asking a client to question their interpretations about an identity-related experience could be extremely invalidating, exasperate distress, and contribute to mistrust of health care providers. Negative interpretations of ambiguous situations can be adaptive in minoritized groups (eg, Black individuals experiencing police brutality), and it is invalidating to suggest reappraising such interpretations. It is not yet known whether similar approaches that focus on cognitive reappraisal, such as interpretation bias interventions, are experienced as invalidating by minoritized individuals, even when not directly focused on racism-related interpretations.

During the development of HabitWorks, we attended to sociocultural identities in several ways. Although HabitWorks presents common situations related to cognitive distortions in anxiety and depression, these same situations could also bring up thoughts related to discrimination. Thus, we first completed a review of the Word Sentence Association Paradigm (WSAP) stimuli to minimize the risk of ambiguous situations bringing up experiences related to microaggressions or discrimination [15]. Two research assistants who identify with multiple minoritized identities examined 800 word-sentence pairs from various versions of the WSAP using the ADDRESSING framework [16]. ADDRESSING is an acronym for sociocultural identities including age, disability, diagnosis status, religion or spirituality, ethnicity or race, sexuality, socioeconomic status, Indigenous heritage, national origin, and gender. Each research assistant selected 1 sociocultural identity at a time and identified word-sentence pairs that strongly corresponded to potentially negative experiences related to that identity. Next, potentially problematic situations were either removed or modified to reduce association with identity-related negative experiences. Third, we collected qualitative feedback from participants via self-report exit questionnaires in all our pilot trials. In all studies, we specifically asked about any negative effects of the WSAP as well as cultural acceptability (eg, “Do you think your friends or family would want to use an app like this?”). Participants did not share any negative experiences related to the WSAP content related to identity-related experiences. Finally, we created a “HabitWorks in context” document in the app that describes the difference between anxiety-related negative interpretations and identity-related interpretations. For example, a person of a minoritized race or ethnicity may draw conclusions about the situation—“You have a job interview”—because of thoughts related to performance anxiety (“I’ll appear nervous and they will think I’m weak”) and racism (“They are going to judge me differently because of my race”). To provide participants with more context, we state that HabitWorks is designed to help people re-evaluate thoughts related to anxiety and depression. It is *not* the intention of HabitWorks to reframe interpretations related to discrimination because it is unhelpful, culturally insensitive, and invalidating to reframe thoughts related to racism, sexism, homophobia, ableism, or any other form of discrimination experienced.

Because modifying interpretations around discrimination-related experiences could be invalidating, we instead provided an extensive catalog of general and identity-specific resources to provide support in finding a therapist, mental health organizations, crisis lines, financial resources, identifying supportive social spaces, and ways to cope with discrimination.

While we hope these initial efforts resulted in an intervention that is safe and acceptable to minoritized groups, we currently do not have sufficient data to determine this. In our prior trials, we did not specifically ask about identity-related experiences in qualitative interviews. In addition, the interviewers were all White, which may impact participants’ comfort in disclosing their experiences. In addition, our small pilot studies did not have a sufficient sample size of participants identifying as Black or Latinx; across the 3 pilot trials, only 2 participants identified as Black and only 3 identified as Hispanic or Latinx. Thus, it is currently unclear how a history of microaggressions and discrimination affect how users experience the HabitWorks app.

### Objectives

This study aims to test safety, acceptability, and engagement with the HabitWorks app in a sample of Black and Latinx adults. On the basis of our prior pilot work, we hypothesized that HabitWorks would be feasible to deliver to a community sample of Black and Latinx adults and that participants would adhere to the recommended dosage. However, we were unsure whether HabitWorks would be safe and acceptable to Black and Latinx participants. To answer this question, we examined the rate of adverse outcomes and conducted qualitative interviews after participants used the app for 1 month. To address the limitations of our prior work, qualitative interviews were all conducted by a person of a minoritized racial or ethnic identity (although not necessarily Black or Latinx). In addition, interviews specifically probed whether HabitWorks brought up experiences of discrimination or was invalidating in any way. We compared safety, feasibility, acceptability, interpretation bias, and anxiety outcomes to a priori benchmarks selected based on prior studies [2,4,17,18]. These prior studies selected benchmarks based on previous interpretation bias modification studies in clinical settings and clinical judgment based on what would be clinically useful for a low-intensity smartphone intervention [4]. Given that this was a similar pilot trial, we used these same benchmarks. Benchmarks were selected before reviewing current data.

## Methods

### Participants

Participants were English-speaking adults aged ≥18 years and residing in the United States, who identified as Black, Hispanic, or Latinx. Additional inclusion criteria included access to an iPhone and at least mild anxiety symptoms (Generalized Anxiety Disorder [GAD]-2 score ≥3) or depression symptoms (Patient Health Questionnaire [PHQ]-2 score ≥3). Exclusion criteria included active mania or psychosis that would inhibit informed consent or completion of study procedures.

**Figure 1:**
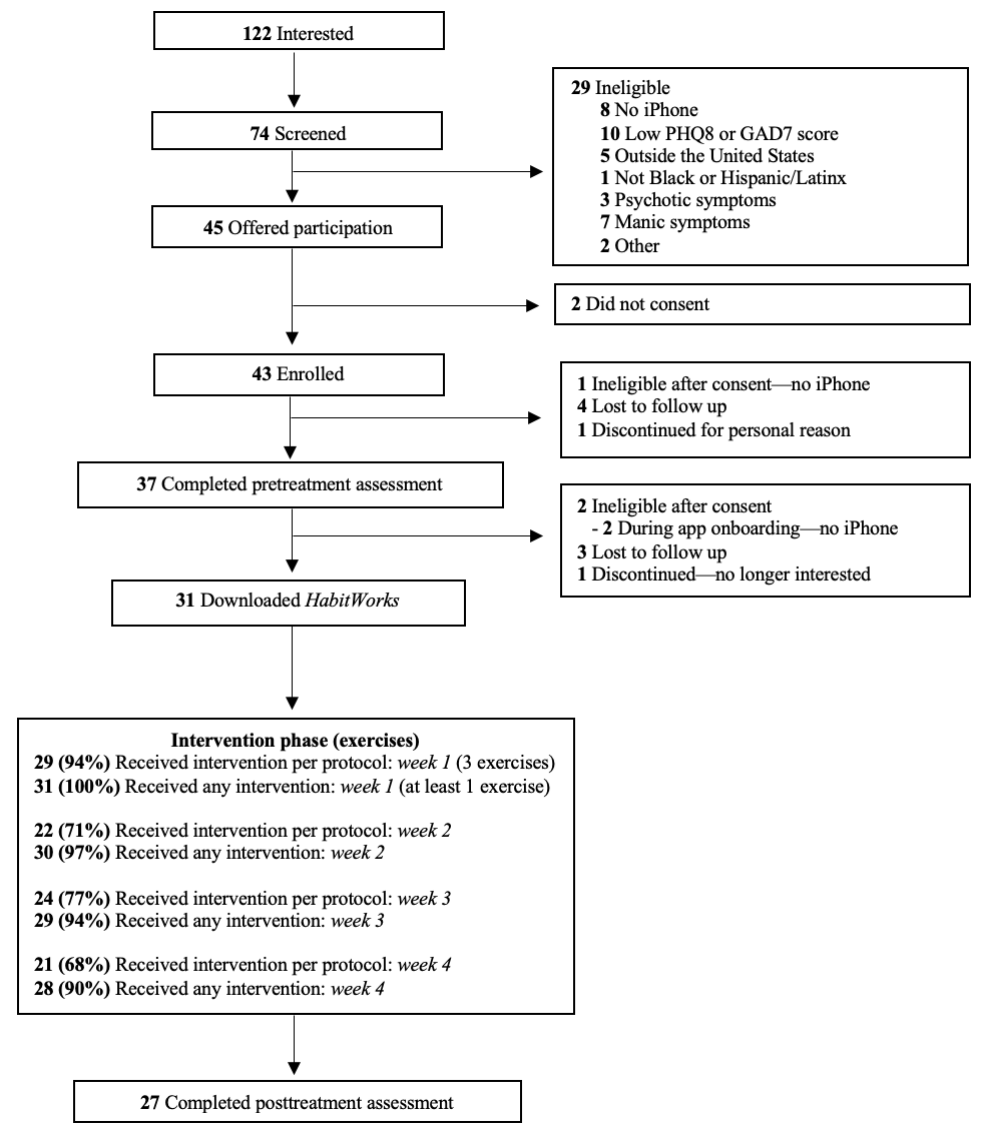
CONSORT (Consolidated Standards of Reporting Trials) diagram. Ineligible subgroups at the screening stage are not mutually exclusive. GAD-7: Generalized Anxiety Disorder; PHQ-8: Patient Health Questionnaire.

The study was posted for 4 months (November 2022 to February 2023) on Mass General Brigham’s Rally website, a web-platform that advertises clinical research studies to the public. Participants were informed that the study was testing a smartphone app designed to reduce anxiety and depression. A total of 31 participants were included in analyses (Figure 1). Participants identified as primarily women (22/31, 71%), with their ages ranging from 18 to 61 (mean 32.83, SD 11.06) years, and most participants completed ≥4 years of college (18/31, 58%) and were employed full time (18/31, 58%; Table 1). Participants identified as Black (15/31, 48%), Latinx (13/31, 42%), and both Black and Latinx (3/31, 10%). Most participants resided in Massachusetts (24/31, 77%); however, we also enrolled 1 participant from each of the following states: Illinois, New York, Nebraska, Connecticut, Rhode Island, Tennessee, and South Carolina.

**Table 1:** Demographic characteristics of participants (N=31).

Characteristic		Participants
Age (y), mean (SD; range)		32.83 (11.06; 18-61)
**Sex at birth, n (%)**		
	Female	23 (74)
	Male	8 (26)
	Intersex	0 (0)
	Prefer not to answer	0 (0)
**Gender, n (%)**		
	Cisgender, woman	22 (71)
	Cisgender, man	7 (23)
	Transgender, man	1 (3)
	Transgender, woman	0 (0)
	Nonbinary	0 (0)
	Genderqueer	0 (0)
	Agender	0 (0)
	Not listed	0 (0)
	Prefer not to answer	1 (3)
**Sexual orientation, n (%)**		
	Asexual	0 (0)
	Bisexual	3 (10)
	Gay or lesbian	3 (10)
	Heterosexual or straight	23 (74)
	Pansexual	1 (3)
	Queer	1 (3)
	Not listed	0 (0)
	Prefer not to answer	0 (0)
**Education level, n (%)**		
	8th grade or less	0 (0)
	Some high school	0 (0)
	High school graduate or General Educational Development credential	1 (3)
	Some college or associate’s degree or trade school	12 (39)
	4-year college graduate	10 (32)
	Postcollege education	8 (26)
	Prefer not to answer	0 (0)
**Employment, n (%)**		
	Student	6 (19)
	Student and employed part time	1 (3)
	Not employed due to disability	1 (3)
	Not employed—seeking job	2 (6)
	Employed part time	2 (6)
	Employed full time	18 (58)
	Employed full time and part-time	1 (3)
	Homemaker	0 (0)
	Retired	0 (0)
**Race and ethnicity, n (%)**		
	American Indian or Alaska Native	0 (0)
	American Indian or Alaska Native, White, Hispanic or Latinx	1 (3)
	Asian	0 (0)
	Black or African American	12 (39)
	Black or African American and Hispanic or Latinx	6 (19)
	Black or African American and White	2 (6)
	Hispanic or Latinx	5 (16)
	Hispanic or Latinx, Native Hawaiian or Pacific Islander, and White	1 (3)
	Hispanic or Latinx and White	4 (13)
	Middle Eastern or North African	0 (0)
	White	0 (0)
	Do not know or unsure	0 (0)
	Not listed	0 (0)
	Prefer not to answer	0 (0)

### Measures

Outcomes for the pilot study included safety, feasibility, acceptability, target engagement (interpretation bias), and anxiety and depression symptoms.

#### Safety

We tracked the number of participants who experienced any adverse outcomes, as well as clinical deterioration, defined as an increase of ≥5 points from the previous assessment on the GAD-7 or PHQ-8 during the 1-month treatment period.

#### Adherence

We prescribed 3 exercises per week for 4 weeks. We obtained exercise completion data from the app user statistics. In addition to the interpretation exercises, HabitWorks prompted participants to complete a self-report assessment of anxiety and depression symptoms and a diary entry weekly. Participants could also complete assessments of their symptoms and diary entries at any point during the treatment phase. We calculated the number of each completed.

#### Acceptability

First, we administered a 10-item self-report measure of participant satisfaction previously used in similar studies [2,4,18]. Administered during the posttreatment assessment time point, this exit questionnaire prompted participants to rate how helpful, relevant, user-friendly, and satisfying they found HabitWorks after 1 month of use on a Likert scale from 1 (*completely disagree*) to 7 (*completely agree*). In addition to the 5 Likert scale response items, the 5 remaining items had an open-ended response format (Multimedia Appendix 1)*.*

Second, participants were asked to complete a semistructured qualitative interview following completion of the 1-month treatment phase via telephone. Interviewers were members of the research team (KON, GG, and HB). Before conducting interviews, all interviewers underwent comprehensive training, including an in-depth reading of relevant articles about qualitative interviews and observing a qualitative interview led by the principal investigator for a related study. The principal investigator developed the initial interview guide, which was then reviewed by experts in mental health in Black and Latinx populations with no further edits suggested. The interviewers then reviewed the guide and suggested some minor wording edits. Interview prompts asked about general impressions of HabitWorks; negative experiences due to the app, including whether it brought up thoughts related to discrimination; the effectiveness of the program; and suggestions for improvement. Interviews (n=20) ranged in duration from 7.68 to 60.65 (mean 19.38, SD 12.18) minutes.

We used a general inductive approach [19], a simple and straightforward procedure that is well suited to summarizing focused evaluation questions such as those in this study. Within the context of the general inductive approach, we applied a rapid analysis procedure, which is preferred for intervention development, implementation, and time-sensitive analyses needed to inform future waves of data collection [20]. Rapid analysis approaches have demonstrated comparable rigor to traditional qualitative analyses with less time and cost [21].

Four members of our team conducted the qualitative analysis (KON, FGH, ZM, and AT). We first created a template representing the domains of inquiry in the interview guide. Each coder independently read each transcript and recorded themes in the template. Each coder then reviewed the template several times to identify an initial set of themes across transcripts. They then met together to discuss their independently derived themes to reduce overlap and redundancy. They then reread transcripts or listened to interview recordings to ensure the existing categories captured all participant data relevant to our study aims as well as to identify contradictory points of view. They met again to discuss any revisions to the themes and decide on the ultimate structure of the data. Finally, the team met to discuss and reach consensus about the themes and structure.

#### Usability

We used the 10-item self-report System Usability Scale to examine participant ratings of the usability of HabitWorks [22]. Administered at the posttreatment assessment time point, the System Usability Scale asked participants to rate how usable (eg, “cumbersome,” “integrated,” and “easy”) they found the intervention, using a Likert scale ranging from 1 (*strongly disagree)* to 5 (*strongly agree)*. Technologies scoring >68 are classified as above average regarding usability [23]. This measure has exhibited excellent reliability and validity [23] and excellent internal consistency in the current sample (Cronbach α=0.94).

#### Credibility and Expectancy

After their first session of HabitWorks, participants were asked to complete the Credibility and Expectancy Questionnaire (CEQ) [24]. The CEQ is a widely used 6-item self-report measure with 2 factors: credibility (items 1 to 3, eg, “How logical does the therapy offered to you seem?”) and expectancy (items 4 to 6, eg, “How much improvement in your symptoms do you really feel will occur?”) designed to assess participants’ thoughts and feelings toward the intervention’s ability to reduce symptoms (ie, stress and anxiety). A rating scale of 1 (not at all) to 9 (completely), or 0% to 100%, is used for each question, depending on question content. The CEQ has demonstrated good test-retest reliability, adequate validity, and good internal consistency [24,25], including in the current sample (Cronbach α=0.84 for credibility and 0.85 for expectancy).

#### Interpretation Bias

As a manipulation check, we included an assessment version of the WSAP [26] at baseline and after treatment. The WSAP is a commonly used measure of interpretation bias with good internal consistency and test-retest reliability across clinical and nonclinical populations [3]. In the assessment version, no feedback is provided about accuracy of responses. The 50 word-sentence pairs were drawn from a previous study of interpretation bias and can be found on the internet [27,28]. Thus, stimuli were not personalized in the assessment version, meaning that all participants saw the same transdiagnostic set of 50 word-sentence pairs. Of note, although there is a chance that some word-sentence pairs were seen in both the intervention and assessment for some participants, overall, the WSAP assessment presented distinct stimuli from the intervention. Participants were asked to decide if a word and sentence were related. Half of the ambiguous sentences were paired with a word reflecting the negative or threat interpretation, and half were paired with a word reflecting a benign interpretation. For example, the word “bored” would reflect the negative or threat interpretation, while the word “tired” would reflect the benign interpretation for the sentence “Someone yawns while you’re talking.” Responses were coded as accurate when participants endorsed “yes—related” to benign trials and “no—not related” to negative trials. We calculated an interpretation bias score by averaging each participant’s accuracy score across the 50 trials; higher accuracy scores reflect less negative interpretation bias. As the WSAP assessment is a similar task to the intervention, we emphasize that this measure served as more of a manipulation check (ie, did participants learn the “correct” contingencies in the intervention and did this generalize to an assessment version of the task?) than a test of change in general interpretative style. The WSAP was administered via a customized link sent to each participant’s email at each time point.

#### Anxiety

Participants completed the GAD-7 item scale during in-app mood check-ins and at baseline and posttreatment assessment time points. The GAD-7 is a widely used 7-item self-report questionnaire that assesses symptoms of anxiety on a Likert scale ranging from 0 (*not at all*) to 3 (*nearly every day*), with higher scores indicating greater anxiety severity [29]. The GAD-7 has demonstrated good reliability and construct validity [30,31] and has been used as a transdiagnostic measure of anxiety in various clinical settings [30,32].

#### Depression

Participants completed the PHQ-8 item scale during in-app mood check-ins and at baseline and posttreatment assessment time points. The PHQ-9 is a widely used self-report questionnaire that assesses depression symptoms on a Likert scale ranging from 0 (*not at all*) to 3 (*nearly every day*), with higher scores indicating greater depression severity [33]. The PHQ-8 includes all items from the PHQ-9 except the ninth item assessing thoughts of death and harming oneself. The PHQ-9 has demonstrated good reliability and construct validity [34], and dropping item 9 of this questionnaire is common practice and has been shown not to affect reliability and validity [35].

### Ethical Considerations

This study was approved by the Mass General Brigham Institutional Review Board (2022P001752). All participants provided informed consent at the beginning of the study and were able to opt out at any time. Privacy and confidentiality were maintained by deidentifying all data. Additionally, the HabitWorks app was designed to minimize risks of breach of privacy, confidentiality and data security. Participants were compensated up to US $100 for their participation. They received US $20 for completing the baseline and posttreatment assessments, US $30 for cellular data use related to the app, and US $30 for completing the feedback interview. Participants were not compensated for using the HabitWorks app, including the interpretation exercises.

### Procedure

Study staff emailed potential participants a link to a brief screening survey to assess initial eligibility via REDCap (Research Electronic Data Capture; Vanderbilt University), a secure, web-based application designed to capture data for research studies [36]. If eligible, participants provided informed consent via REDCap. Once consented, participants were asked to complete a pretreatment assessment via REDCap. Following the pretreatment assessment, participants met with the research assistant via Zoom (Zoom Video Communications) to complete a 30-minute orientation session. During this session, the participants downloaded the HabitWorks app, watched instructional videos explaining the rationale of and how to use HabitWorks features, completed the HabitWorks personalization checklists, scheduled notifications to complete WSAP exercises, and practiced completing the first symptom survey and WSAP exercise in the app.

Participants were asked to use the HabitWorks smartphone app for 1 month. During this month, we asked them to complete the WSAP exercises 3 times per week, a symptom survey weekly, and a Habit diary entry weekly. The Habit diary encouraged participants to write about their progress by prompting them to write about instances when they jumped to a conclusion or noticed a change in their thinking behavior. Refer to the study by Beard et al [4] for an in-depth description of app features, and refer to the study by Beard et al [2] for a detailed description of the app development process.

The WSAP exercises took approximately 5 minutes to complete and included feedback that reinforced benign interpretations after each trial. Specifically, participants saw “Correct!” when they endorsed a benign interpretation or rejected a negative interpretation. They saw “Try again!” when they rejected a benign interpretation or endorsed a negative interpretation. Participants completed 50 trials during each scheduled exercise and 30 trials during any user-initiated bonus exercise. HabitWorks presented participants with personally relevant ambiguous situations from a pool of 714 potential word-sentence pairs, based on their responses to personalization checklists. Refer to the study by Beard et al [4] for more information on personalization.

A research assistant monitored adherence and emailed participants weekly. These weekly emails summarized the participant’s progress that week and offered encouragement. If the intervention was not completed that week, the email provided support related to low motivation and technological issues. In addition, the research assistant was available to meet with participants via Zoom or phone to provide technical support, although no participants asked to meet. After 1 month of app use, participants were sent a posttreatment assessment via REDCap*.* Once the posttreatment assessment was completed, participants were invited to complete a feedback interview.

## Results

### A Priori Benchmarks

A priori benchmarks and obtained data are presented in Table 2.

**Table 2:** Benchmarks.

Outcome	Target	Actual
Safety	No clinical deterioration or increase in suicidal ideation possibly related to study participation	0 participants reported worsening due to study participation
Feasibility (% eligible provided consent)	50% of eligible participants provide consent	43/45, 95%
Adherence to WSAP^a^ exercises	50% complete sessions 3 times per week during the treatment phase	Week 1: 29/31, 93%; week 2: 29/31, 71%; week 3: 24/31, 77%; and week 4: 21/31, 68%
Adherence to study assessments	75% complete assessments	Baseline: 37/43, 86% and posttreatment: 27/31, 87%
Credibility	At least moderate credibility (5 on a scale of 1 to 9 for the CEQ^b^ item “How logical?”)	Mean 6.19, SD 2.15
Expectancy	At least moderate expectancy (>50% out of 100% on the CEQ item “How much improvement?”)	Mean 45.56%, SD 24.2%
Satisfaction and acceptability	Average rating of “slightly agree” (5 on a scale of 1 to 7) on the exit questionnaire	Helpfulness: mean 5.19, SD 1.44; relevance: mean 4.78, SD 1.63; easy to use: mean 6.15, SD 1.23; satisfaction: mean 5.78, SD 1.50; and recommendation: mean 5.46, SD 1.63
Interpretation bias manipulation check	75% in healthy range (≥70% accuracy) on WSAP (note that 1/31, 3% were already in the healthy range at baseline)	Posttreatment: 23/31, 74%
Symptom reduction	50% in the “none to minimal” range on GAD-7^c^; effect size Cohen *d* >0.2	Posttreatment: 9/31, 29%; Cohen *d*=0.41

^a^WSAP: Word Sentence Association Paradigm.

^b^CEQ: Credibility and Expectancy Questionnaire.

^c^GAD-7: Generalized Anxiety Disorder-7.

### Safety

During the 4-week treatment period, no adverse events were reported. Overall, 16% (5/31) of the participants were flagged for clinical deterioration; 4 (80%) of these 5 participants demonstrated clinical deterioration once, while 1 (20%) participant demonstrated it twice. None of these were deemed related to the HabitWorks intervention or research procedures.

### Feasibility

Of the 45 participants eligible for the study, 43 (95%) enrolled. Of the 43 enrolled participants, 31 (72%) downloaded the app. All recruitment, assessment, and intervention procedures were easily completed entirely remotely. Participants did not report any substantial issues with using the app.

### Adherence

**Figure 2:**
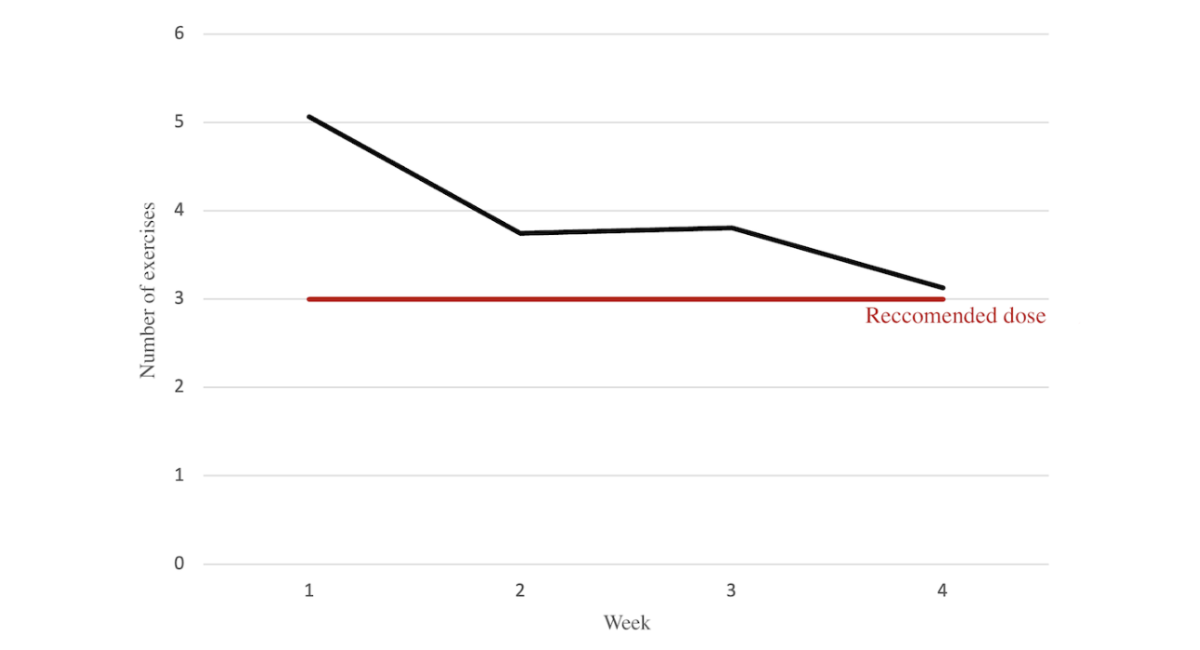
Average number of Word Sentence Association Paradigm (WSAP) exercises completed in the treatment phase (N=31).

On average, participants completed 15.74 (SD 7.43) exercises in total out of the 12 prescribed over the 4 weeks. Of the 31 participants who onboarded to the app, 17 (55%) adhered to the prescribed intervention (3 exercises/wk) all 4 weeks, and 27 (87%) participants used the intervention all 4 weeks (Figure 2). Approximately two-thirds (21/31, 68%) of the participants completed at least 1 symptom survey every week of their participation. On average, participants completed 6.13 (SD 3.35) symptom surveys by the end of the 4 weeks. Overall, 29% (9/31) of the participants completed a diary entry each week, and 74% (23/31) of the participants completed at least 1 entry during their participation. On average, participants completed 3.55 (SD 3.77) entries out of the 4 prompted with highest completion rates occurring at the end of week 2.

### Acceptability

Acceptability ratings met our a priori benchmarks for satisfaction, perceived helpfulness, user-friendliness, and willingness to recommend the app to a friend (Table 2). A priori benchmarks for relevancy were not met.

Data from qualitative interviews also revealed high acceptability and few negative experiences (Multimedia Appendix 1). Regarding participants’ general experience with HabitWorks, themes such as easy to use, intuitive, quick, and efficient emerged. People commented on several aspects of the app that they found helpful, such as the notifications, mood check-ins, exercises, and progress tracking graphs. Far fewer people discussed unhelpful aspects of HabitWorks, and these centered on the exercises feeling tedious or repetitive, the words flashing too quickly, and some instructional content being confusing. When asked about any negative experiences, most people denied any; a few people reported feeling briefly frustrated when the app told them they were incorrect about a situation, or they did not score as high as they wanted. When asked about any perceived changes since using HabitWorks, participants observed changes in their cognition, such as reappraising situations and not jumping to conclusions. Participants discussed increased awareness of their thought patterns and mood. They also noted changes in their emotions, such as feeling less anxious.

We were very interested in the personal relevance of the situations. Overall, participants reported that the situations presented in the exercises were frequently experienced and relatable to their daily life. However, a few participants (5/19, 26%) also noted specific situations that they did not find personally relevant. We also specifically asked participants whether the situations in the WSAP exercises triggered any thoughts of discrimination, racism, or microaggressions. Participants overwhelmingly did not think the WSAP brought up experiences of discrimination and denied any race-related distress while completing it. However, 2 participants did report that some of the situations could be related to xenophobia, social exclusion, and work-related discrimination.

### Usability

On average, participants reported that HabitWorks had good usability (mean 67.60, SD 27.96; range 10.00-100.00), just approaching the cutoff of 68 as an indicator of above average usability [23].

### Credibility and Expectancy

On average, participants reported that HabitWorks was moderately credible (mean 6.13, SD 2.19; range 1 to 9). On average, participants reported that their expectation for percent improvement in their mental health symptoms was 43% (SD 22.77%; range 10%-100%) and did not meet our a priori benchmark.

### Interpretation Bias

**Figure 3:**
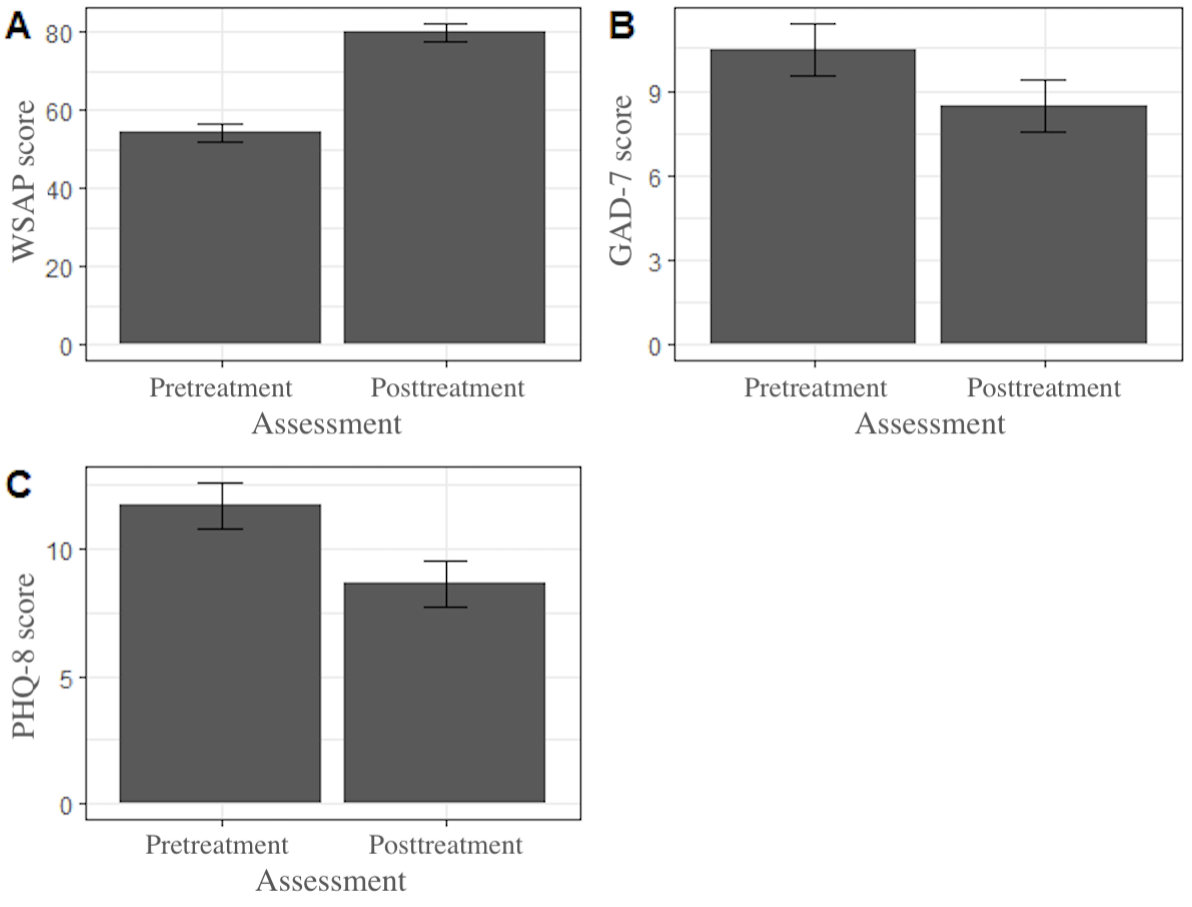
(A) Word Sentence Association Paradigm (WSAP), (B) Generalized Anxiety Disorder-7 (GAD-7), and (C) Patient Health Questionnaire-8 (PHQ-8) scores (N=31).

Five participants did not complete the WSAP at the posttreatment time point. Given the small pilot sample, we carried forward these participants’ baseline scores for this time point. Our a priori benchmark for the assessment version of the WSAP was met (Table 2). At baseline, 3% (1/31) exceeded the healthy score threshold for accuracy (≥70% accuracy). By the posttreatment assessment, 74% (23/31) of the participants’ interpretation bias scores on the WSAP task were in the healthy range. A paired samples 2-tailed *t* test revealed significant improvement in accuracy (t_30_=2.29; *P*<.001; Figure 3). The effect size was large with a Cohen *d* of 1.53.

### Anxiety and Depression Severity

Overall, 100% (31/31) of the participants completed the PHQ-8 and GAD-7 at baseline, and 87% (27/31) of the participants completed the PHQ-8 and GAD-7 at the posttreatment time point. We carried forward the last reported score on the PHQ-8 and GAD-7 for the 4 participants missing their postassessment. At baseline, the average GAD-7 score was in the “moderate” severity range (mean 10.47, SD 4.69). The average GAD-7 score reduced to the “mild” severity range at the posttreatment time point (mean 8.48, SD 5.66). A paired samples *t* test revealed a significant decrease in anxiety symptom severity (t_30_=2.29; *P*=.03; Figure 3). The effect size was small with a Cohen *d* of 0.41. At baseline, the average PHQ-8 score was in the “moderate” severity range (mean 11.71, SD 4.53). The average PHQ-8 score reduced to the “mild” severity range (mean 8.65, SD 5.64). A paired samples *t* test revealed a decrease in depression symptom severity (t_30_=3.065; *P*=.005; Figure 3). The effect size was medium with a Cohen *d* of 0.55.

### Suggestions for Improvement

Suggestions to improve HabitWorks included ideas to improve exercises such as increasing the number of situations or adding the ability to change difficulty level. In addition, participants felt exercises could be better understood by giving participants an opportunity to explain their answers or offering explanations for incorrect answers. There were some participants (6/19, 32%) looking for enhancement of esthetic and (5/19, 26%) improvements to the Habit diary. Participants also mentioned wanting app features that offered more support such as human support through the app (eg, coach), additional strategies for learning healthy habits and coping skills, and links to other resources. Overall, participants expressed satisfaction with the app and wanting to share it with others (Table 2).

## Discussion

### Summary

We tested the preliminary safety, feasibility, and acceptability of a smartphone-delivered interpretation bias intervention in a sample of adults who identify as Black, Hispanic or Latinx, or both. This study addresses a critical gap, given that prior studies of DMHIs and HabitWorks*,* specifically, have not had sufficient representation of Black or Latinx participants. HabitWorks met almost all a priori benchmarks for safety, feasibility, adherence, and acceptability.

### Safety, Feasibility, and Acceptability

Overall, the evidence of safety, feasibility, and acceptability of HabitWorks for Black or Latinx adults was found to be strong and consistent with previous studies of HabitWorks comprising primarily non-Hispanic White participants [4,5]. No clinical deterioration occurred due to the use of the app. The a priori benchmark for the credibility of HabitWorks was met. Satisfactions ratings were high; participants found HabitWorks to be helpful and easy to use and would recommend the app to a friend. The a priori benchmarks not met were treatment outcome expectancies, as found in previous trials testing HabitWorks [4,5]. We suspect that expected improvement in mental health symptoms was low due to skepticism around DMHIs, as treatment-seeking individuals often initially feel that DMHIs are inferior in comparison to other mental health care options such as counseling and medications [37-40]. Future research should consider how education on the efficacy of DMHIs in comparison to other treatments could boost expectancy. For instance, we could educate users on the high satisfaction ratings of HabitWorks during the onboarding process to try to address low expectancy.

Despite personalizing the situations presented, our benchmark for personal relevancy was not met, and data from qualitative interviews also revealed that some participants did not find all the scenarios to be personally relevant. Engagement and adherence were good despite this, but suboptimal relevancy might explain the decrease in use of the app after the first week. This could partially be due to the limited variety in word-sentence pairs, which some participants mentioned during qualitative interviews. To achieve our relevance benchmark, we may need to increase the number of word-sentence pairs presented and provide more personalization questions to narrow the scope of scenarios provided within the exercises. It is also possible that incorporating participant-generated situations would improve relevancy as well as engagement. Despite the room for improvement in personalization of content, overall satisfaction was good.

It is also important to note that although expectancy for HabitWorks was low at baseline, overall satisfaction with the app across several domains was high at the posttreatment time point, as in previous studies of HabitWorks [4,5]. This was supported by qualitative data, which found that some participants wanted to continue to use the app beyond study participation. Therefore, experience using the app seemed to overcome initial skepticism and led to acceptability of the app allowing for higher engagement and adherence rates.

One of our main interests in this study was determining whether the intervention content was culturally sensitive and whether the exercises brought up experiences of racism or discrimination. Although we attended to sociocultural identities when developing HabitWorks*,* it was not culturally adapted for Black or Latinx communities. Despite the lack of specific cultural adaptation, HabitWorks demonstrated comparable acceptability to other culturally adapted DMHIs [12]. Overall, participants did not feel that HabitWorks asked them to reframe experiences of discrimination or caused them any race-related distress. When asked, only 2 participants reported that some situations brought up thoughts about xenophobia, feeling excluded, and work-related discrimination. Distress related to these situations was not reported and could have been attenuated by the previously mentioned app features such as the “HabitWorks in context” document and the ability for participants to remove word-sentence pairs they did not want to see again. Relatedly, as noted previously, we intentionally did not include any situations in HabitWorks that that could bring up identity-related stress. Thus, the current version of HabitWorks was not designed to address uncertainty and stress due to identity-related experiences. Because some minoritized individuals may have daily negative experiences related to their identity, there are many important research questions for future work. For example, it is possible that shifting participants’ general interpretive style might generalize to identity-relevant situations and provide some anxiety reduction in identity-related situations. Thus, future studies should include measures of identity-related stress. It is also possible that entirely different types of interventions are better suited to help people cope with ambiguous situations that bring on identity-based stress; thus, future studies might compare the effects of interpretation bias interventions combined with specific identity-based stress coping interventions. Overall, these findings suggest that HabitWorks shows promise as a culturally appropriate DMHI for Black and Latinx adults.

### Adherence and Engagement

We asked participants to complete 3 WSAP exercises per week for 1 month. On average, participants exceeded this and completed 15.74 WSAP exercises in the 1-month period, with 54% (17/31) of the participants completing the prescribed in intervention all 4 weeks. Most people stop using mental health apps after 2 weeks [41]; however, HabitWorks use remained high even in week 4. As previously noted, some participants even expressed a desire to continue using HabitWorks after completing the study. Moreover, participants engaged with the other features of the app. On average, participants completed 6.13 symptom surveys in a 1-month period, with most participants completing at least 1 symptom survey every week, demonstrating high engagement with the symptom survey feature. Habit diary completion rates fell just short of the recommended dose of 1 per week. Qualitative data revealed mixed opinions of the Habit diary, as some participants found it helpful, while others did not and offered suggestions for its improvement (Multimedia Appendix 1). Importantly, we did not compensate participants for using the app, and most participants enrolled immediately before the winter holiday season. Thus, we find these adherence rates encouraging, especially considering the typical challenges of engaging users, particularly during this time of year. However, compensation for cellular data use and completion of the feedback interview may have acted as motivation for participants to complete the study.

A meta-analysis revealed that culturally adapted DMHIs have an average attrition rate of 42%, despite reporting high satisfaction [12]. In contrast, this pilot trial demonstrated high satisfaction and a low attrition rate, with 67% (21/31) of the participants meeting the recommended treatment dose by the end of the study. These adherence rates provide strong, preliminary evidence that this intervention has the potential to be an engaging treatment for anxiety and depression across races and ethnicities.

### Interpretation Bias

We examined interpretation bias before and after treatment. On average, participants’ interpretation bias significantly improved from baseline to the posttreatment time point with a large effect size. By the end of treatment, 74% (23/31) of the participants had moved in the healthy range, which is slightly lower than our a priori benchmark and what was found in other studies testing this type of intervention [2,4,18]. These findings are promising in that they demonstrate an ability for participants to learn from the correctional feedback provided by the WSAP exercises and apply it to new situations, but without additional measures of interpretation bias, we cannot demonstrate an improvement in interpretation bias that translates beyond the task itself. However, we can draw upon participants’ perceived cognitive changes. In qualitative interviews, participants reported that HabitWorks increased their awareness of jumping to conclusions, and their ability to reappraise situations had improved with app use.

### Anxiety and Depression Severity

We also examined anxiety and depression symptom severity before and after treatment. On average, participants’ depression and anxiety symptom severity significantly decreased from baseline to the posttreatment time point, with small to medium effect sizes, respectively. Participants also expressed in qualitative interviews that they felt better and less anxious. Given that this study was an open trial without a control group, we cannot determine what drove this symptom improvement, as it could be due to HabitWorks or due to expectancy, attention from researchers, or other factors. However, this degree of symptom improvement is encouraging and suggests that further testing is warranted.

### Limitations

Although this study addresses important limitations of prior studies testing DMHIs, we note several limitations that are inherent in a pilot open trial. First, this study was not designed to test efficacy and therefore included a small sample and did not include a comparison arm. Second, most of our sample was, relatively, highly educated and employed full time, limiting our understanding of the feasibility of HabitWorks for populations with lower income. Third, we prioritized qualitative data to gain a deeper understanding of participants’ experience; however, results from this small sample may not generalize to the entire Black and Latinx populations. The Hispanic and Latinx communities are extremely diverse, and we were unable to examine outcomes for specific groups withing this community (eg, Mexican Americans and Puerto Ricans). Finally, at the time of this trial, HabitWorks was only available in English and in iOS, which also limits the generalizability. After securing additional funding, we have now developed the Android version, and creating a Spanish version will be essential for future evaluations of HabitWorks in the Hispanic community.

### Conclusions

This study adds to the scarce literature evaluating DMHIs in Black and Latinx adults. These preliminary findings support the next stage of effectiveness testing in these populations. Future studies should compare HabitWorks to a credible control arm and include an independent measure of interpretation bias to determine whether improvement on the WSAP generalizes to other measures of interpretive style. In addition, HabitWorks should be investigated in other minoritized racial and ethnic groups that were not examined in this study, such as Asian and Asian Americans, to increase generalizability in our findings. Although none of our participants reported negative experiences due to using the app, it will be important to monitor safety in future studies with larger samples.

According to the 2020 census, only 5.08% of psychologists are African American or Black and 7.95% are Hispanic or Latinx, despite the US population identifying as 13.6% Black and 19.1% Hispanic or Latinx [42,43]. Moreover, waitlists for treatment services with a mental health provider across racial and ethnic identities are extremely long, hindering access to care. DMHIs, such as HabitWorks, have the potential to help bridge the gap between the demand for mental health services and low availability of providers, while addressing the most common barriers, such as cost, stigma, childcare, and time away from work. Our study provides evidence that HabitWorks may be a beneficial culturally sensitive and responsive intervention for minoritized racial and ethnic groups.

## Appendix

Multimedia Appendix 1 Qualitative feedback themes.

## Abbreviations

CEQ: Credibility and Expectancy Questionnaire
DMHI: digital mental health intervention
GAD: Generalized Anxiety Disorder
PHQ: Patient Health Questionnaire
REDCap: Research Electronic Data Capture
WSAP: Word Sentence Association Paradigm
